# Supplementary material for: NOCICEPTRA: Gene and microRNA Signatures and Their Trajectories Characterizing Human iPSC‐Derived Nociceptor Maturation
Source: Adv Sci (Weinh). 2021 Sep 5;8(21):2102354. doi: 10.1002/advs.202102354 (PMC8564443; doi:10.1002/advs.202102354)
Supplement: Supplementary file 1 — Supporting Information [file ADVS-8-2102354-s001.pdf]

© 2021 Wiley-VCH GmbH

Supporting Information

for *Adv. Sci.*, DOI: 10.1002/advs.202102354

NOCICEPTRA: Gene and microRNA signatures and their trajectories characterizing human iPSC-derived nociceptor maturation

Maximilian Zeidler, Kai K. Kummer, Clemens L. Schöpf, Theodora Kalpachidou, Georg Kern, M. Zameel Cader and Michaela Kress**

Supplementary Figure Legend:

Supplementary Figure 1: (A) Hierarchical clustering of gene expression of each individual cell line per timepoint. **(B)** Intersection analysis of differential expressed genes are visualized in the Venn Diagram for AD2, AD3 and 840 clones. **(C)** WGCNA analysis report, with scale free topology and soft threshold parameters as well as mean and median connectivity
(D) Expression trajectories of the neuropeptides (TAC1, CALCA and CALCB)**. (E)** Immunostaining of CGRP (CALCA, red), peripherin (PER, green), DAPI and the respective linescan-scatterplot at D36 indicating colocalization of CGRP and PER in the cytoplasm and neurites. **(F,G)** Literature described murine nociceptor and developmental marker trajectories in iDNs. **(H)** Temporal trajectories of human DRG enriched transcription factors as described in Ray et al. (2018). **(I)** Spearman correlation analysis of log2 TPMs for iDNs and human cultured DRG (hDIV) and native human DRG (hDRG) obtained from Wangzhou et al. (2020). **(J,K)** Temporal trajectories of cultured human DRG enriched kinases and g-protein coupled receptors, illustrated by means of TPM.

Supplementary Figure 2: (A) Triple label immuofluorescence stainings of SCN7A (Nav1.7), TAC1 (Substance-P), NTRK1-3 (panTRK) and PIEZO2 costained with peripherin, or Tuj1 and DAPI at D16, D26 and D36 during iDN differentiation in AD3 and 840 cells. **(B)** Fluorescence intensity of the markers was measured within the respective field of view and compared between the timepoints. **(C)** Distribution analysis of positively and negatively stained cells within the field of view and comparison between the individual differentiation days. iDN differentiations.

Supplementary Figure S3 (A) Triple labeling immunofluorescence staining using either NANOG or KI-67 together with peripherin (PER) and DAPI .Arrowheads in the upper pannel indicate positively stained NANOG/KI-67 cells at D0. Arrowheads in the lower panel indicate NANOG/KI-67 negative cell cohorts at D36. IF positive cells for NANOG and KI-67 were compared to all DAPI positive cells at D0 and D36 and stacked barplots generated.
(B) Triple labeling of TMEM119 or GFAP together with TUJ1 and DAPI at D36 of iDN differentiation. **(C)** Temporal trajectories of GFAP and TMEM199 indicated low (TMEM119) and absent expression of GFAP

Supplementary Figure 4: (A) Current-voltage relationship accessed for two celllines (AD2, AD3, n = 15 cells) by means of a step-protocol (-60mV – 40mV) and peak as well as sustained current calculated and normalized against the membrance capacitance. **(B)** Resting membrane potential was evaluated in current clamp mode with a 0 pA injected current over 1 minute and three intervals (start (mean_1), middle (mean_2), end (mean_3) selected. **(C)** All cells functionally accessed, were capable of firing action potentials and a representative figure made (n = 10 (AD3)/ 11 (AD2) cells). **(D)** Action potentials were fitted by means of the integrated fitting function implemented in HEKA Fitmaster and Action Potential Duration, Onset as well as duration from onset till repolarization (mindT) investigated. **(H)** Differential expression analysis of voltage-gated sodium channels at D36 between AD3 and AD2

Supplementary Figure 5 - Gene Trajectories: Z-scored standardized gene expression trajectories for each individual module, for each cell line (blue: 840, red: AD2, green: AD3, respectively). X-axis represent differentiation dates, y-axis z-standardized vst-counts

Supplementary Figure 6 - Gene Trajectories: Z-scored standardized gene expression trajectories for each individual module, for each cell line (blue: 840, red: AD2, green: AD3, respectively). X-axis represent differentiation dates, y-axis z-standardized vst-counts.

Supplementary Figure 7 - Gene Trajectories: Z-scored standardized gene expression trajectories for each individual module, for each cell line (blue: 840, red: AD2, green: AD3, respectively). X-axis represent differentiation dates, y-axis z-standardized vst-counts.

Supplementary Figure 8 (A,B) PCA and hierarchical clustering of miRNA expression for each individual cell line. **(C)** Intersection analysis of differential expressed miRNAs are visualized in the Venn Diagram for AD2, AD3 and 840 clones. **(D)** WGCNA parameter report for soft threshold power, median and mean connectivity and max connectivity. Soft threshold topology model fit reaching 0.9 is achieved after the soft-threshold was set to 7. **(E)** WGCNA module preservation reports revealed high preservation across most modules detected with an average preservation score of 0.87. **(G)** qPCR validation of 5 miRNAs (n = 3/timepoint/cellline) enriched at distinct differentiation stages.

Supplementary Figure 9 - miRNA curves: Z-scored standardized miRNA expression trajectories for each individual WGCNA miRNA module for each cell line (blue: 840, red: AD2, green AD3).

Supplementary Figure S10 (A) Temporal Trajectories of FGFR1-3 using vst standardized counts and tpm indicative of relative expression. **(B)** Temporal expression of WNT related genes using vst counts and tpm indicative of relative expression. **(C)** Temporal expression of WNT related genes highly expressed around D9 using vst counts and tpm indicative of relative expression

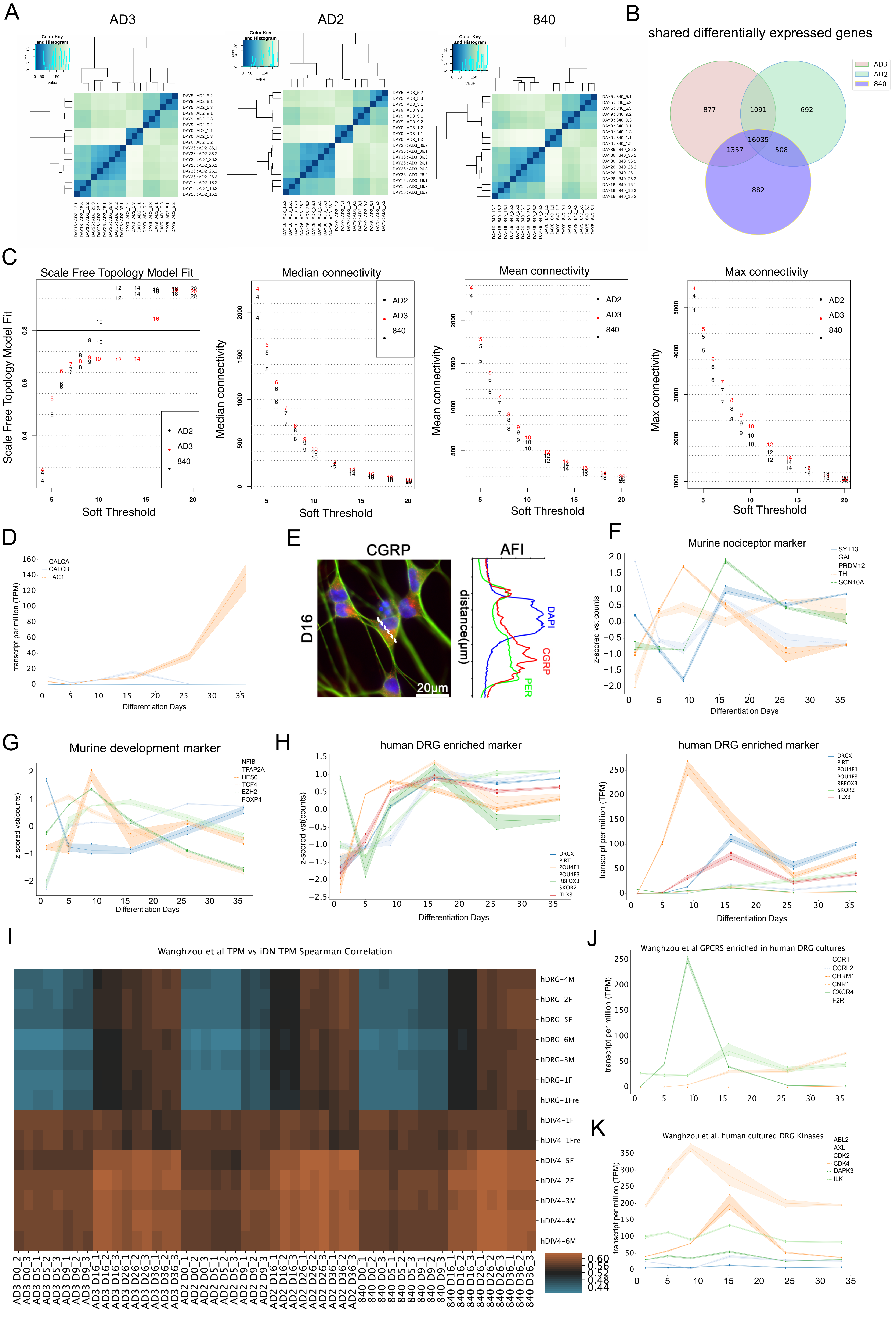

A
AD3
AD2
840
Color Key and Histogram
B
shared differentially expressed genes
AD3
AD2
840
877
1091
692
16035
1357
508
882
C
Scale Free Topology Model Fit
Median connectivity
Mean connectivity
Max connectivity
Scale Free Topology Model Fit
Median connectivity
Mean connectivity
Max connectivity
Soft Threshold
D
CALCA
CALCB
TAC1
transcript per million (TPM)
Differentiation Days
E
CGRP
AFI
D16
20μm
distance(μm)
DAPI
CGRP
PER
F
Murine nociceptor marker
SYT13
GAL
PRDM12
TH
SCN10A
z-scored vst counts
Differentiation Days
G
Murine development marker
NFIB
TFAP2A
HES6
TCF4
EZH2
FOXP4
z-scored vst(counts)
Differentiation Days
H
human DRG enriched marker
DRGX
PIRT
POU4F1
POU4F3
RBFOX3
SKOR2
TLX3
z-scored vst(counts)
transcript per million (TPM)
Differentiation Days
I
Wanghzou et al TPM vs iDN TPM Spearman Correlation
hDRG-4M
hDRG-2F
hDRG-5F
hDRG-6M
hDRG-3M
hDRG-1F
hDRG-1Fre
hDIV4-1F
hDIV4-1Fre
hDIV4-5F
hDIV4-2F
hDIV4-3M
hDIV4-4M
hDIV4-6M
J
Wanghzou et al GPCRS enriched in human DRG cultures
CCR1
CCRL2
CHRM1
CNR1
CXCR4
F2R
transcript per million (TPM)
K
Wanghzou et al. human cultured DRG Kinases
ABL2
AXL
CDK2
CDK4
DAPK3
ILK
transcript per million (TPM)

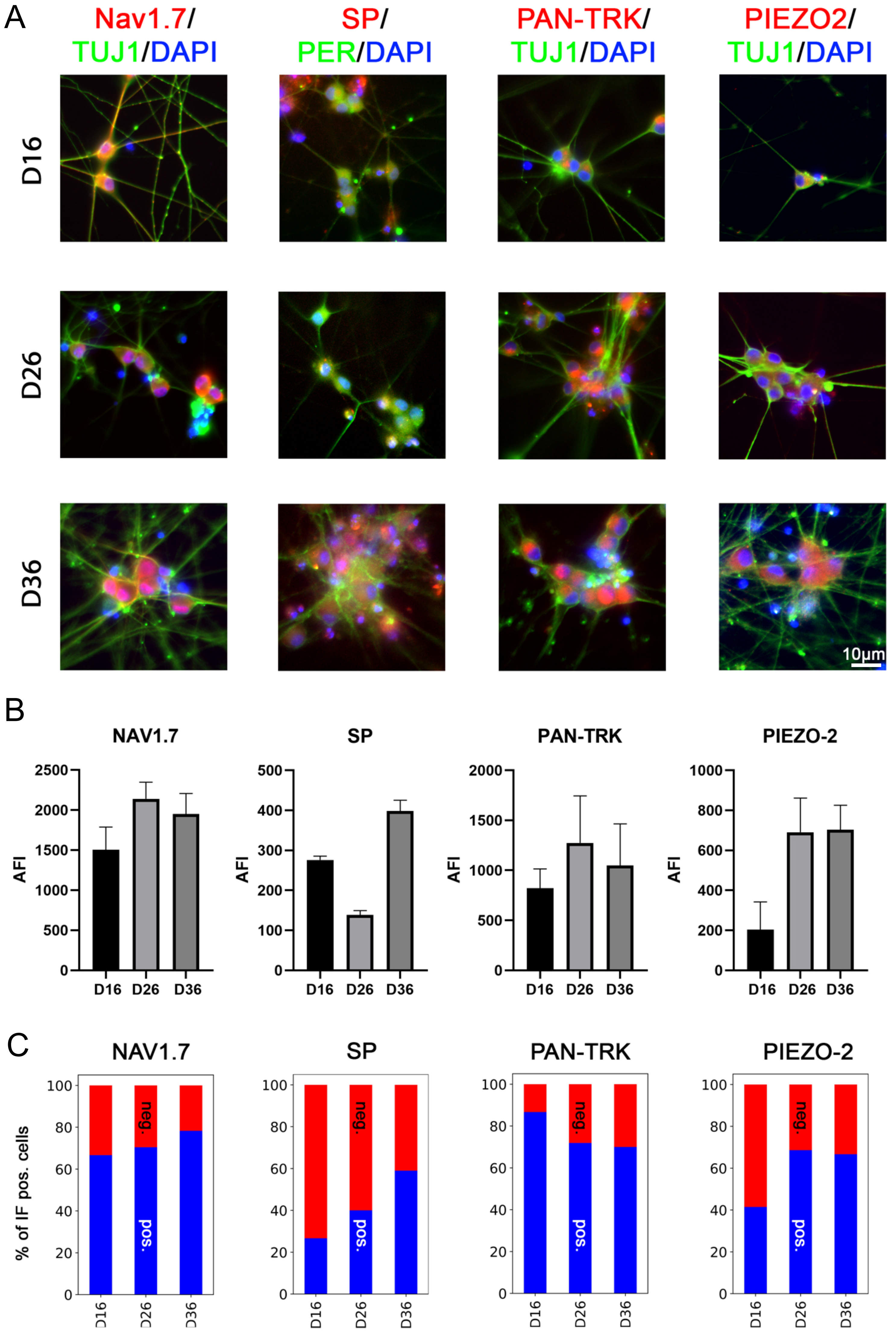
A
Nav1.7/TUJ1/DAPI
SP/PER/DAPI
PAN-TRK/TUJ1/DAPI
PIEZO2/TUJ1/DAPI
D16
D26
D36
10μm
B
NAV1.7
SP
PAN-TRK
PIEZO-2
AFI
D16 D26 D36
C
NAV1.7
SP
PAN-TRK
PIEZO-2
% of IF pos. cells
neg.
pos.
D16 D26 D36

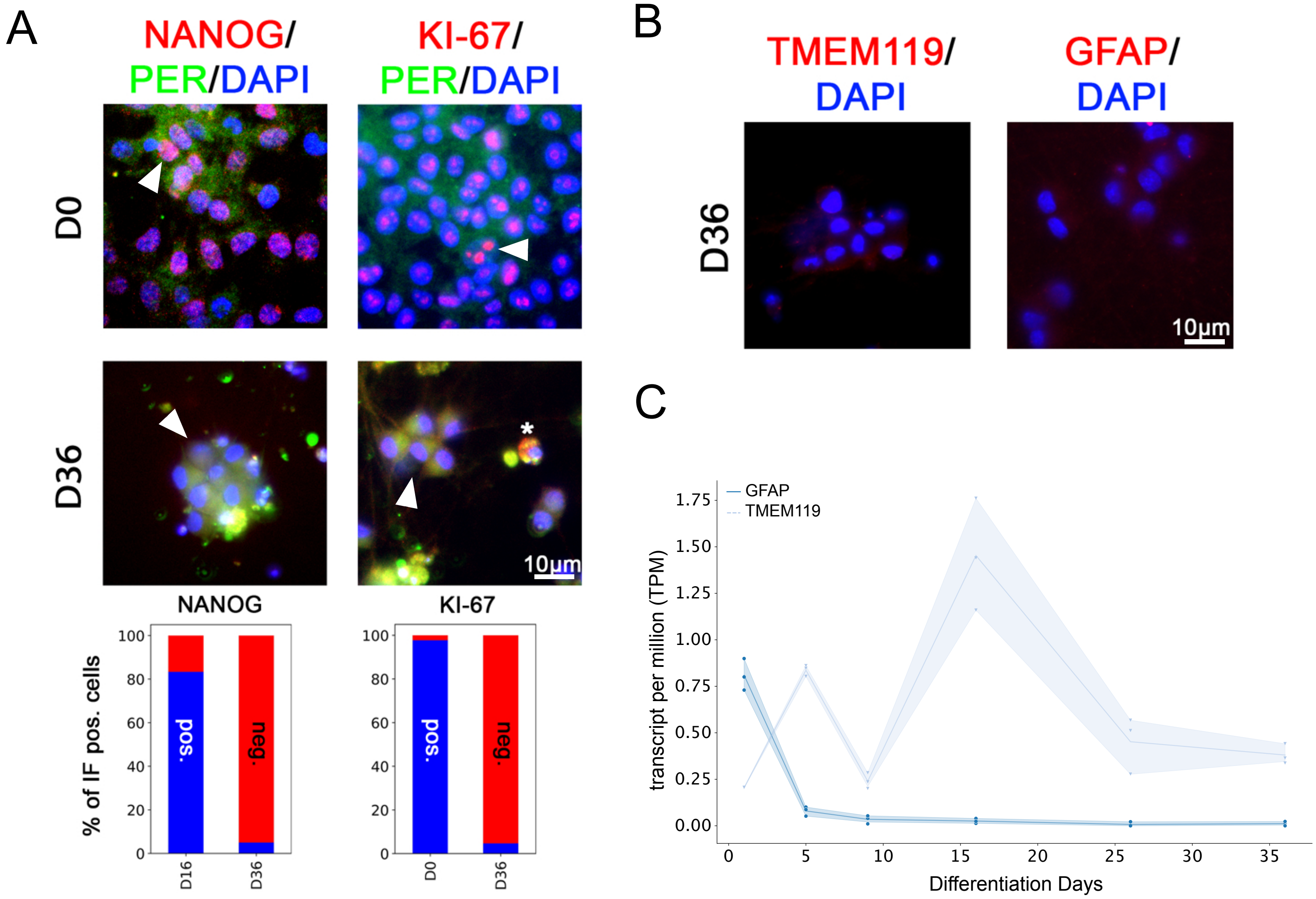
A
NANOG/PER/DAPI
KI-67/PER/DAPI
D0
D36
10µm
NANOG
KI-67
% of IF pos. cells
pos.
neg.
D16
D36
D0
B
TMEM119/DAPI
GFAP/DAPI
C
GFAP
TMEM119
transcript per million (TPM)
Differentiation Days

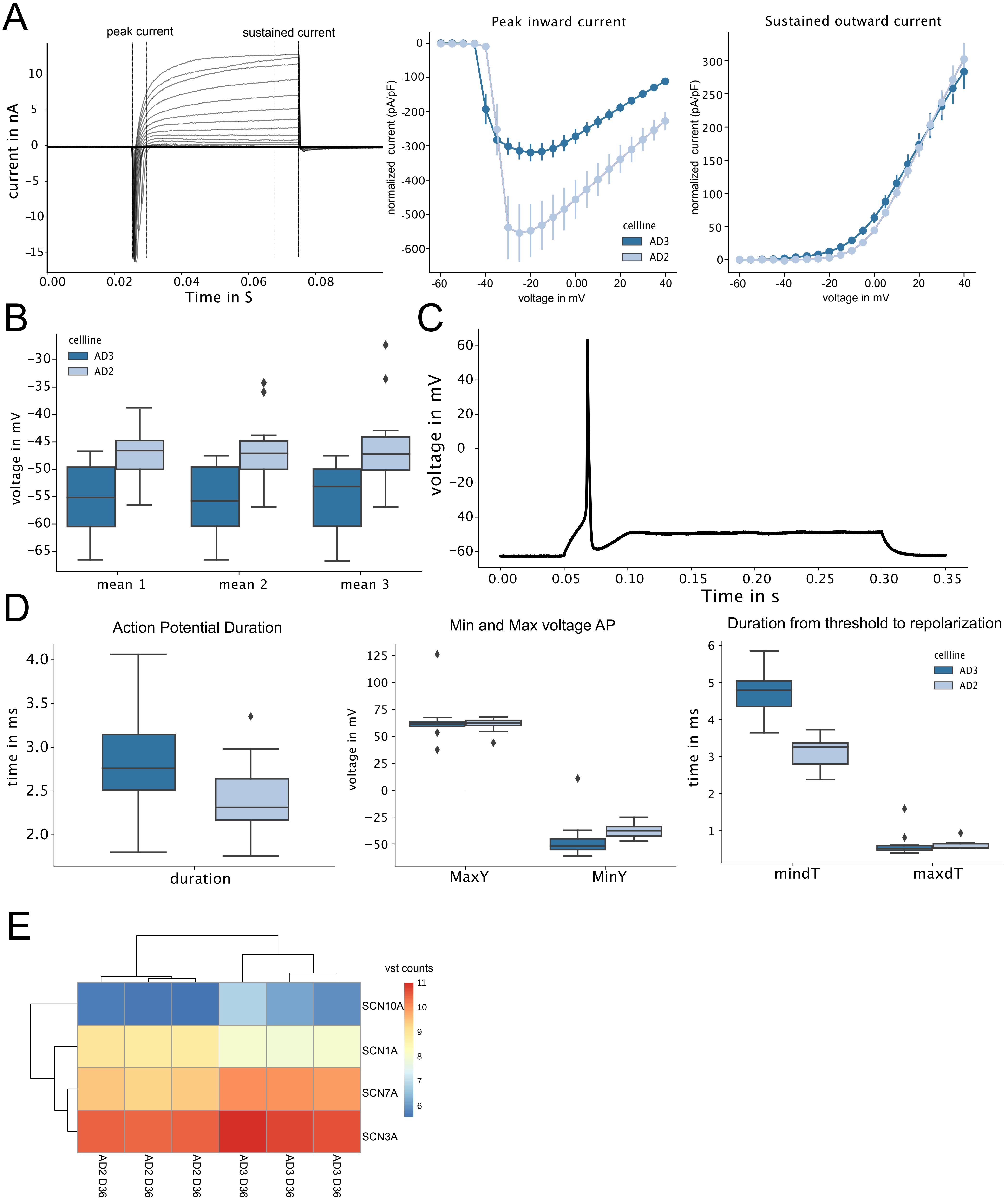

A
peak current
sustained current
current in nA
Time in S
Peak inward current
normalized current (pA/pF)
voltage in mV
cellline
AD3
AD2
Sustained outward current
normalized current (pA/pF)
voltage in mV
B
cellline
AD3
AD2
voltage in mV
mean 1
mean 2
mean 3
C
voltage in mV
Time in s
D
Action Potential Duration
time in ms
duration
Min and Max voltage AP
voltage in mV
MaxY
MinY
Duration from threshold to repolarization
cellline
AD3
AD2
time in ms
mindT
maxdT
E
vst counts
SCN10A
SCN1A
SCN7A
SCN3A
AD2 D36
AD2 D36
AD2 D36
AD3 D36
AD3 D36
AD3 D36

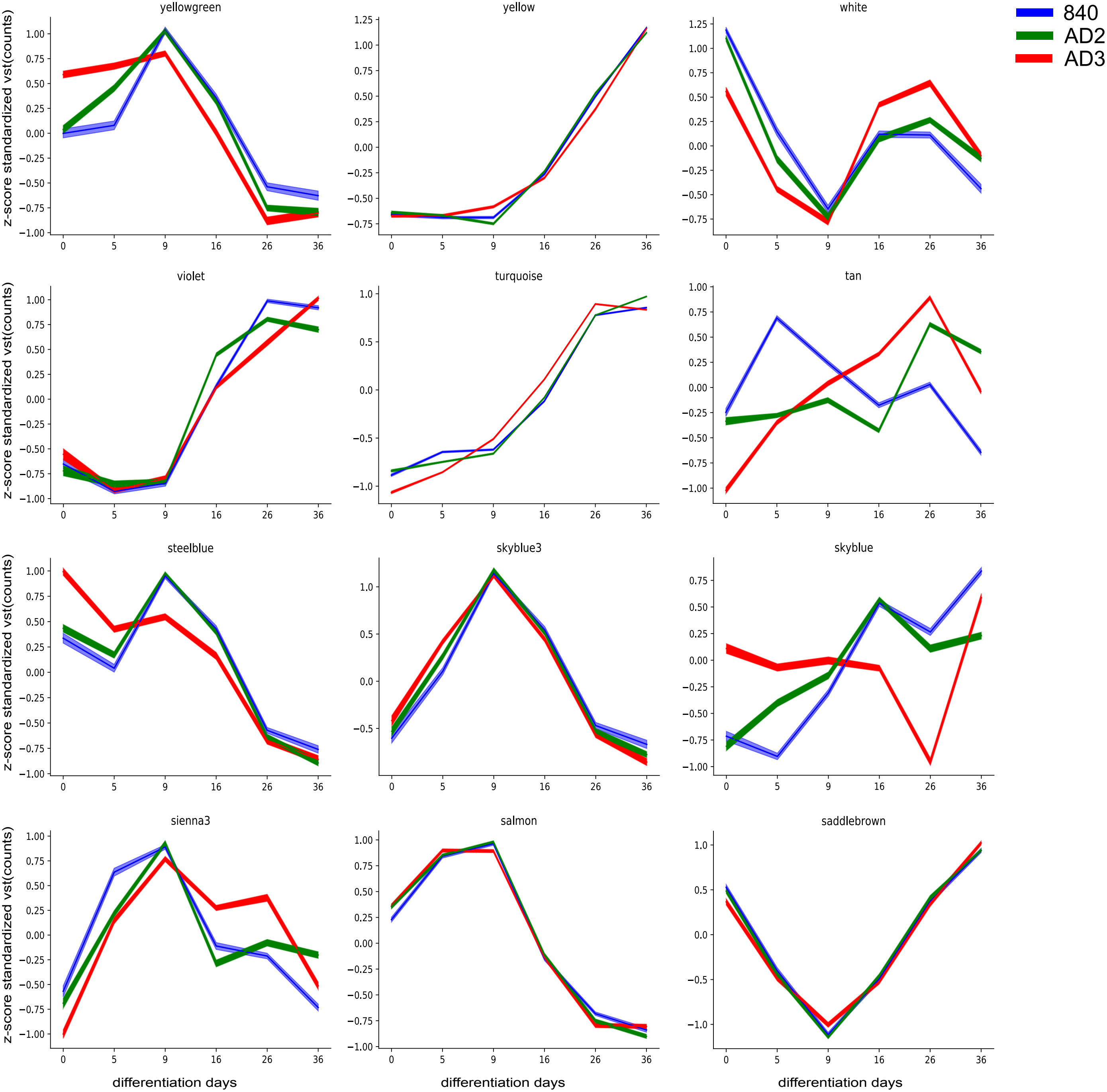
840
AD2
AD3
yellowgreen
yellow
white
violet
turquoise
tan
steelblue
skyblue3
skyblue
sienna3
salmon
saddlebrown
z-score standardized vst(counts)
differentiation days

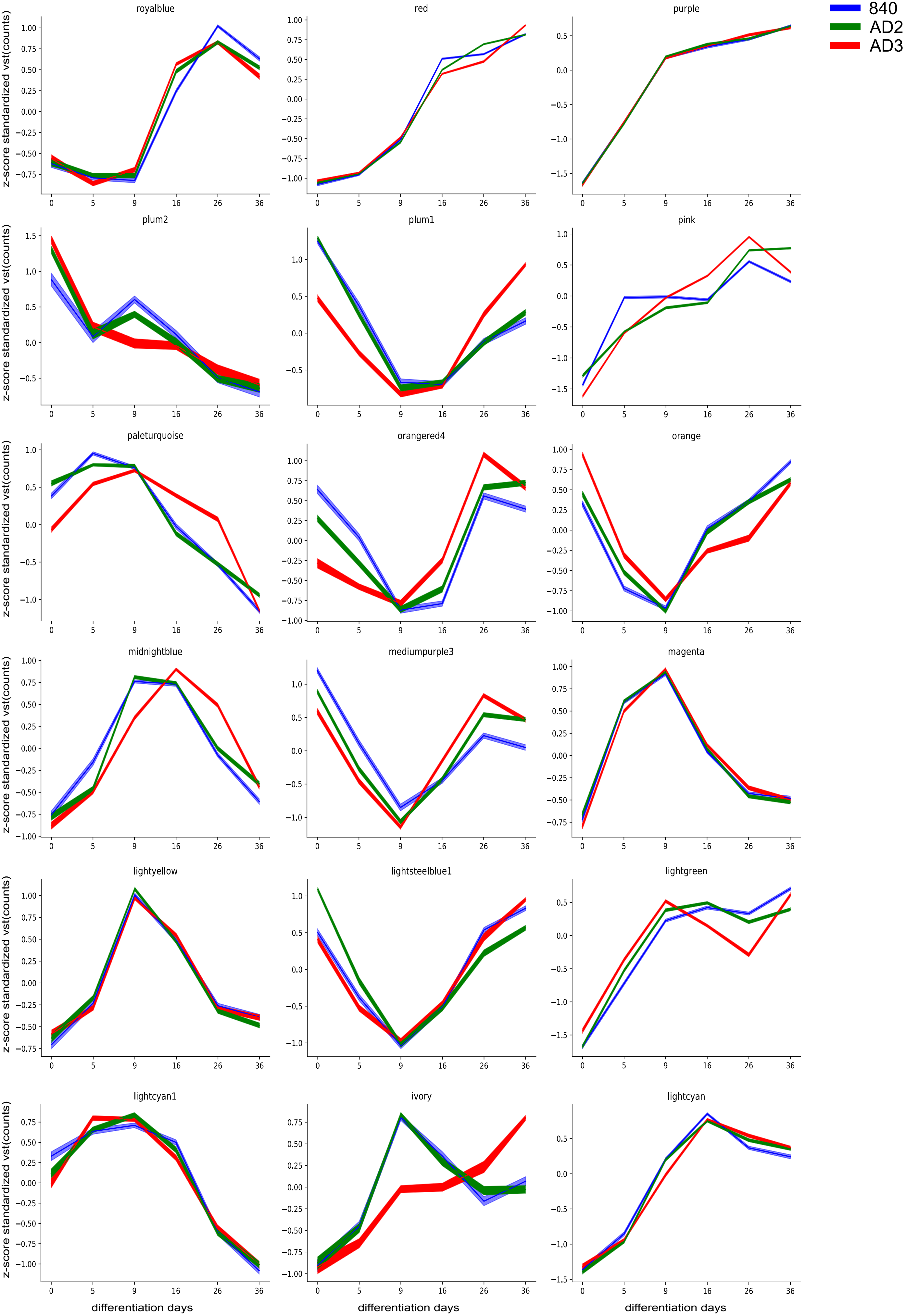
840
AD2
AD3
royalblue
red
purple
plum2
plum1
pink
paleturquoise
orangered4
orange
midnightblue
mediumpurple3
magenta
lightyellow
lightsteelblue1
lightgreen
lightcyan1
ivory
lightcyan
z-score standardized vst(counts)
differentiation days

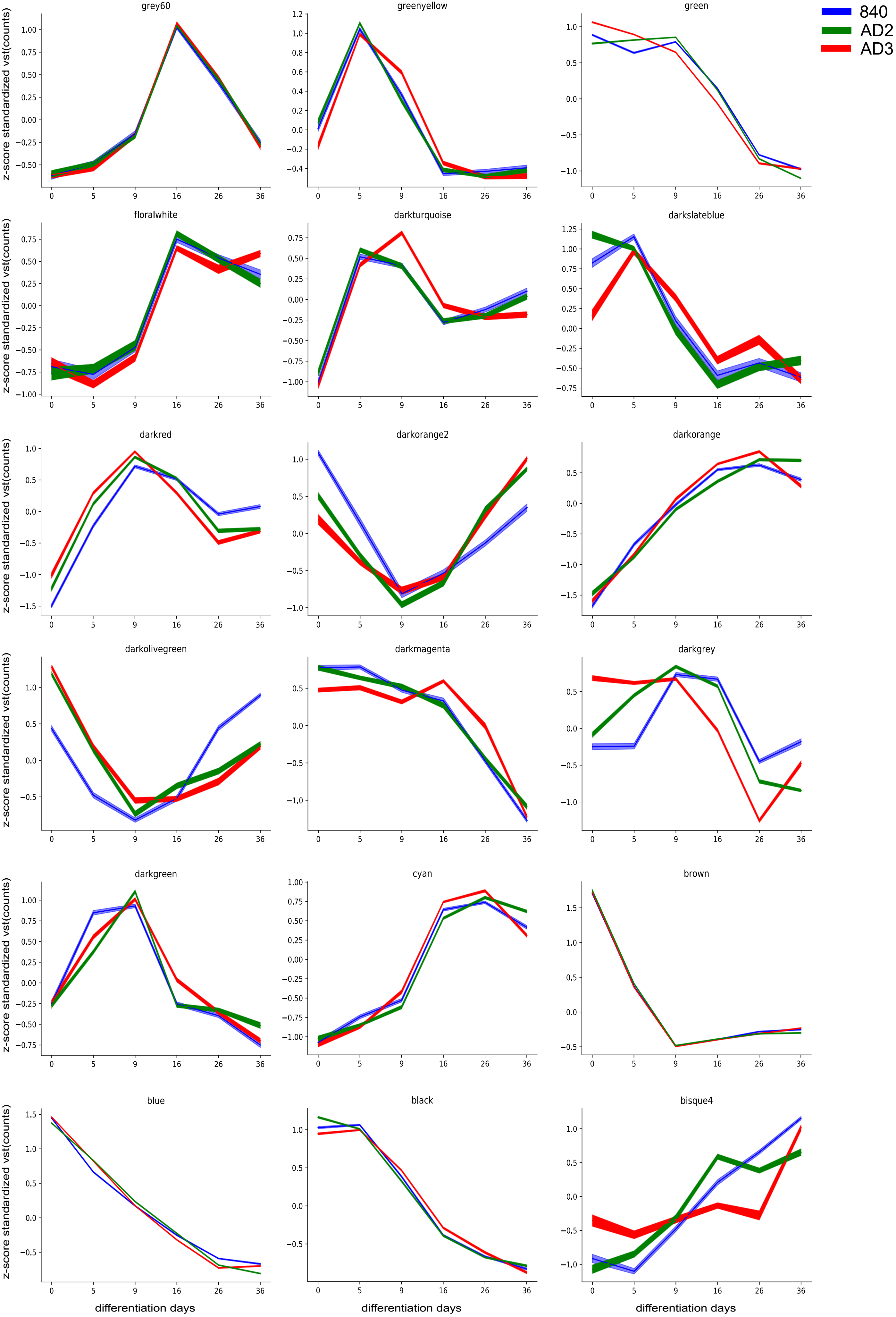
840
AD2
AD3
grey60
greenyellow
green
floralwhite
darkturquoise
darkslateblue
darkred
darkorange2
darkorange
darkolivegreen
darkmagenta
darkgrey
darkgreen
cyan
brown
blue
black
bisque4
z-score standardized vst(counts)
differentiation days
0
5
9
16
26
36

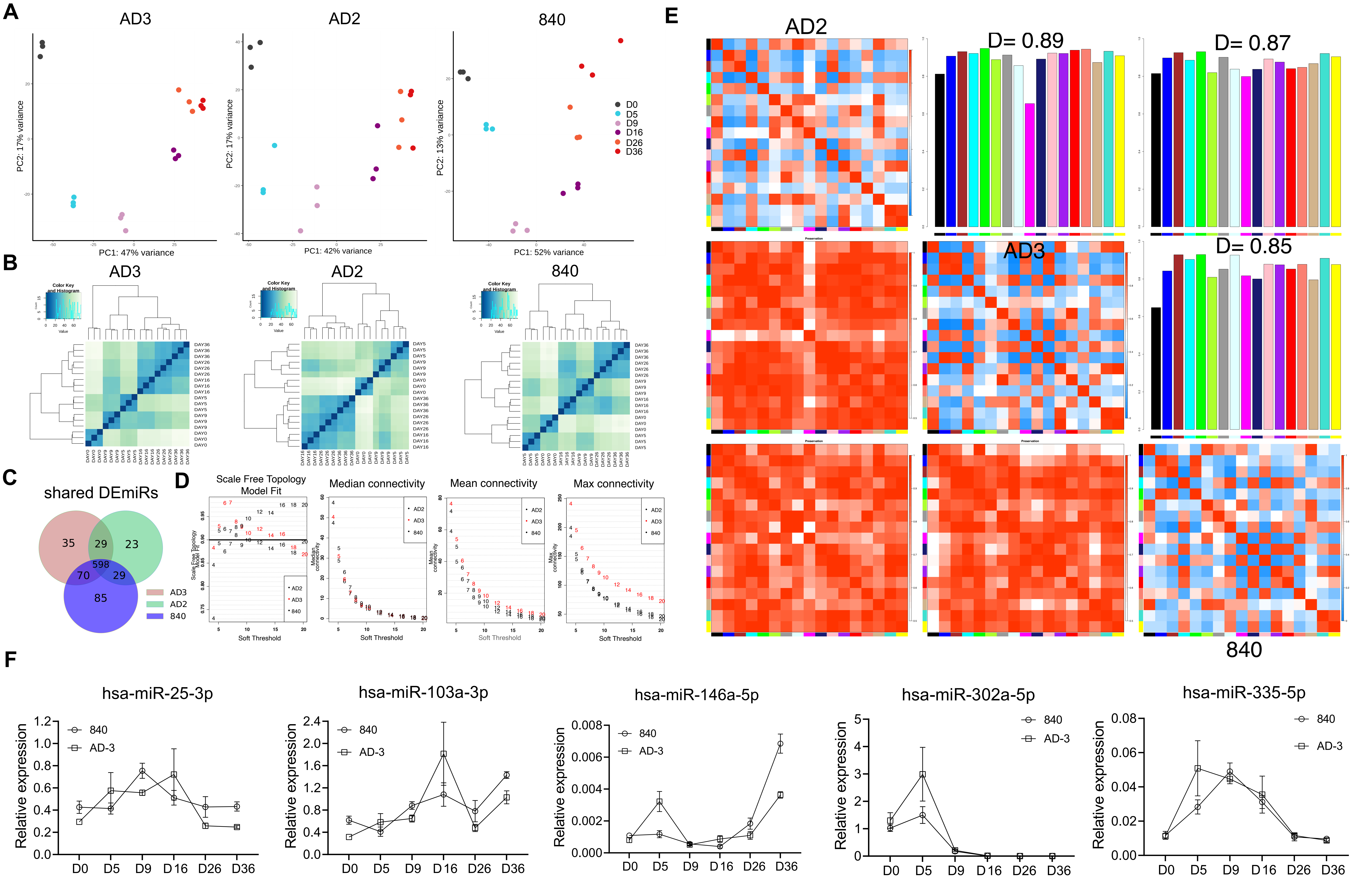
A
AD3
AD2
840
PC1: 47% variance
PC2: 17% variance
PC1: 42% variance
PC2: 17% variance
PC1: 52% variance
PC2: 13% variance
D0
D5
D9
D16
D26
D36
B
AD3
AD2
840
Color Key and Histogram
C
shared DEmiRs
35
29
23
598
70
29
85
AD3
AD2
840
D
Scale Free Topology Model Fit
Median connectivity
Mean connectivity
Max connectivity
Soft Threshold
E
AD2
D= 0.89
D= 0.87
AD3
D= 0.85
840
F
hsa-miR-25-3p
hsa-miR-103a-3p
hsa-miR-146a-5p
hsa-miR-302a-5p
hsa-miR-335-5p
Relative expression
840
AD-3
D0
D5
D9
D 16
D26
D36

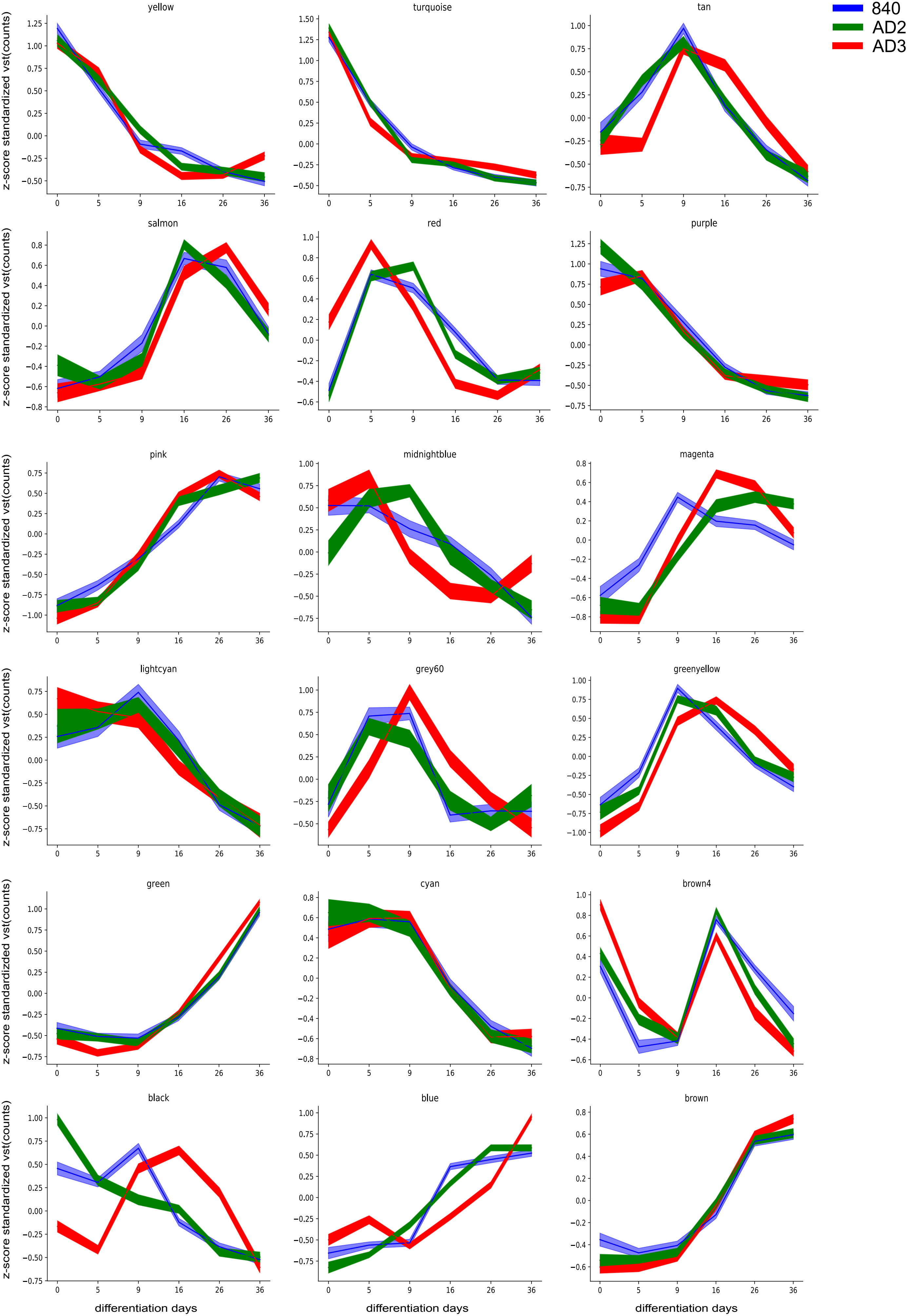
840
AD2
AD3
yellow
turquoise
tan
salmon
red
purple
pink
midnightblue
magenta
lightcyan
grey60
greenyellow
green
cyan
brown4
black
blue
brown
z-score standardized vst(counts)
differentiation days

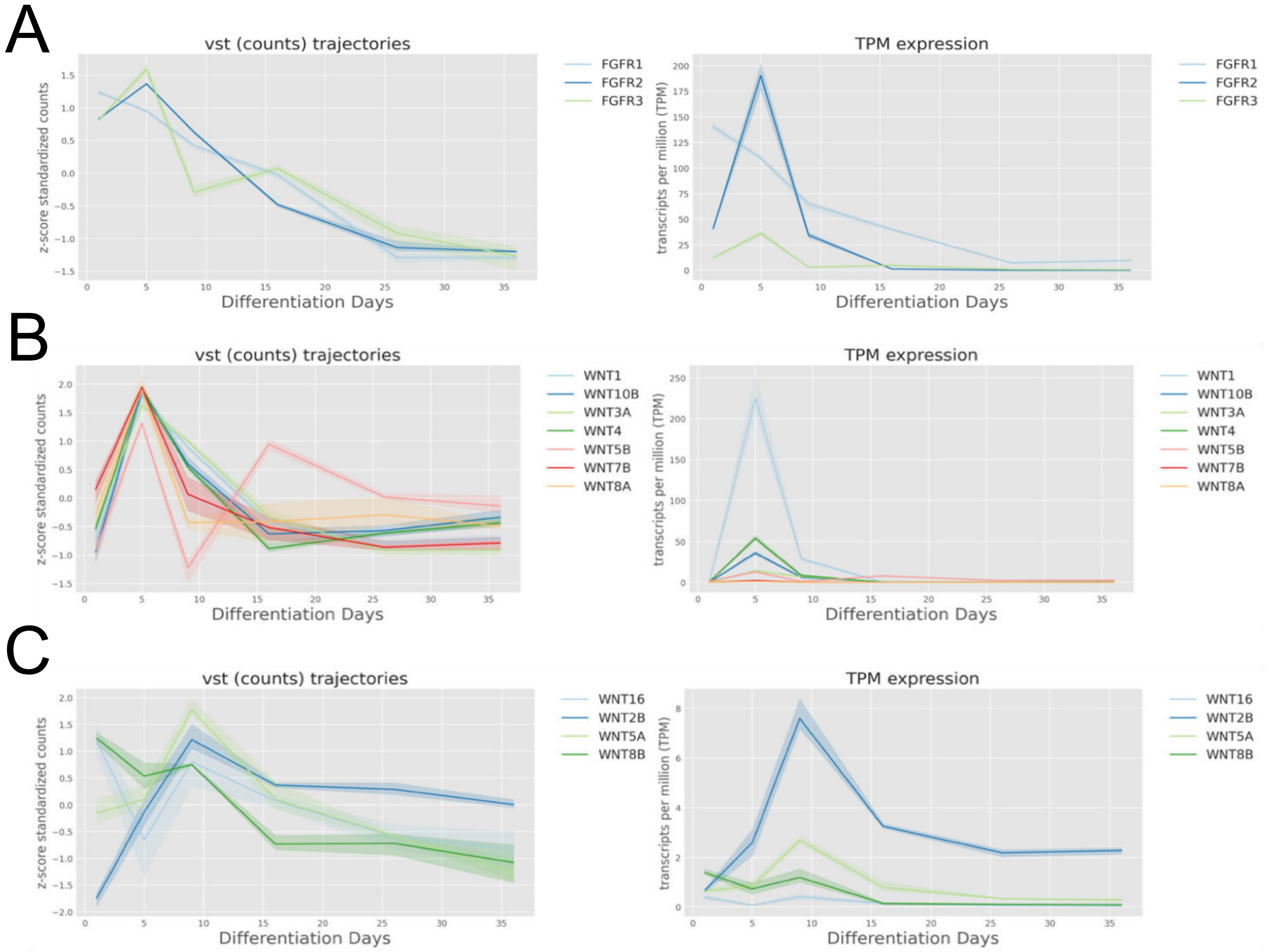
A
vst (counts) trajectories
z-score standardized counts
Differentiation Days
FGFR1
FGFR2
FGFR3
TPM expression
transcripts per million (TPM)
Differentiation Days
FGFR1
FGFR2
FGFR3
B
vst (counts) trajectories
z-score standardized counts
Differentiation Days
WNT1
WNT10B
WNT3A
WNT4
WNT5B
WNT7B
WNT8A
TPM expression
transcripts per million (TPM)
Differentiation Days
WNT1
WNT10B
WNT3A
WNT4
WNT5B
WNT7B
WNT8A
C
vst (counts) trajectories
z-score standardized counts
Differentiation Days
WNT16
WNT2B
WNT5A
WNT8B
TPM expression
transcripts per million (TPM)
Differentiation Days
WNT16
WNT2B
WNT5A
WNT8B
